# Does Treating Hearing Loss in Older Adults Improve Cognitive Outcomes? A Review

**DOI:** 10.3390/jcm9030805

**Published:** 2020-03-16

**Authors:** Hélène Amieva, Camille Ouvrard

**Affiliations:** Inserm U1219 Bordeaux Population Health Center, Université de Bordeaux, 33076 Bordeaux, France; camille.ouvrard-brouillou@u-bordeaux.fr

**Keywords:** hearing loss, hearing aid, cochlear implant, cognitive decline, dementia

## Abstract

Hearing loss is the third most prevalent health condition in older age. In recent years, research has consistently reported an association between hearing loss and mental health outcomes, including poorer cognitive performances. Whether treating hearing loss in elders improves cognition has been directly or indirectly addressed by several studies. This review aims at providing a synthesis of those results. Regarding the literature on hearing aids’ use and cognition, although the lack of interventional studies has to be underlined, observational data suggest that hearing aids positively impact long-term cognition, even though more research is necessary to ascertain this statement and provide information on the length or frequency of use required in order to observe benefits. Regarding cochlear implants in elders experiencing more severe auditory deprivation, the literature is scarcer. The available studies have many limitations and do not allow the drawing of clear conclusions. Taken together, the results are encouraging. Nevertheless, because hearing loss is suspected to account for 9% of dementia cases, and also because hearing loss is one of the few potentially modifiable factors from a dementia prevention perspective, the need to stimulate research to have clearer knowledge of the benefits of treating hearing loss on cognitive outcomes is urgent.

## 1. Hearing Loss and Age-Related Cognitive Decline: Two Highly Frequent Conditions in Aging

The global increase in life expectancy is obviously one of the major health achievements of the past 150 years, since, for the first time in history, most people worldwide can expect to live into their sixties and beyond. The now well-known counterpart of this is that the number of age-related chronic conditions that negatively affect independence and life participation has also substantially increased, contributing to a pessimistic picture of old age. The challenge is not only to live longer, but to live longer with no or little incapacities. Among the numerous conditions which affect the independence and life participation of older adults, hearing loss is one the most common. Age-related hearing loss, also called presbycusis, is a progressive, bilateral and symmetrical multifactorial disorder of hearing sensitivity due the consequence of the aging auditory system, including peripheral and central hearing [1]. It typically results from loss of hair cells at the basilar membrane, primarily affecting high-frequency hearing. As many as one-third of adults aged over 65 report hearing loss, making hearing loss the third most prevalent chronic health condition in older age [2,3]. In recent years, scientific research consistently highlighted the association between hearing loss in elders and numerous mental health outcomes, among which depressive symptoms have been frequently reported, as concluded by a recent meta-analysis of 35 studies, where hearing loss in older adults was associated with an increased risk of experiencing depression [4]. Moreover, hearing loss has been associated with self-perceived older age [5], social isolation [6], reduced daily living activity participation [7,8], and lower quality of life [9,10].

Beyond well-being and psychosocial outcomes, another major domain of mental health suspected to be impacted by hearing loss is cognitive functioning. There is now strong and consistent evidence that older adults experiencing hearing loss present a poorer cognitive performance. Not only have cross-sectional associations between hearing loss and low cognitive scores been reported [11,12,13] but also higher rates of cognitive decline. Indeed, numerous studies involving longitudinal follow-up of participants reported an accelerated cognitive decline with advancing age in older adults suffering from hearing impairment compared to those with no hearing loss [14,15,16,17,18,19,20,21,22,23], results that have been updated and synthesized in several review papers [24,25,26]. With such a body of evidence on the link between hearing loss and accelerated age-related cognitive decline, the question of whether hearing loss increases the risk of developing neurocognitive disorders arises. First, as is well known, in the coming years, along with a life expectancy increase, the number of people suffering from neurocognitive disorders is expected to increase. Secondly, although the causes of most dementing illnesses remain to be clarified, the physio-pathological pathways (accumulation and deposition of beta-amyloid for Alzheimer’s disease or vascular neurodegeneration for vascular dementia) are assumed to be modulated by a wide range of factors—including psycho-social, life-style, nutritional factors—that contribute to moderate, either positively or negatively, the incidence and clinical expression of symptoms. Therefore, the question of whether hearing loss increases the risk of developing dementia when one ages appears to be particularly relevant. These last 5 years, a number of studies involving a longitudinal design provided valuable input. Although still limited in number, these studies report consistent results, supporting the hypothesis of an increased risk of dementia which remains significant even after statistically accounting for potential confounders such as demographic and/or cardiovascular factors. Such results have been gathered from studies considering hearing loss either with audiometric measures [15,27,28], speech recognition tests [29], or indirect/self-reported measures [30,31,32]. Although most of the studies considered peripheral hearing loss, the link between age-related central auditory processing disorder [33] and cognitive decline and/or dementia has also been reported by two longitudinal studies [34,35].

## 2. Possible Mechanisms Explaining the Relationship Between Hearing Loss and Cognitive Decline in Older Adults

To explain the relationship between hearing loss and cognitive impairment, the literature has proposed several potential mechanisms. Two recent articles [36,37] offer a quite thorough review of it, so these hypotheses will be only briefly mentioned in the present paper.

A first, relatively practical, explanation is that hearing impairment may bias the cognitive testing procedure, often involving verbal tests or orally provided test instructions. For instance, if a list of to-be-remembered words is orally given to a participant with hearing loss, the participant may require additional effort to accurately perceive the words, with less cognitive resources being available for encoding/learning processes. Future studies should make use of assessment tools suitable for people with a hearing impairment. It is noteworthy that two such tools have been recently developed: the Repeatable Battery for the Assessment of Neuropsychological Status for Hearing-Impaired individuals (RBANS-H) [38] providing audiovisual presentation of the instructions and the test items and the Hearing-Impaired Montreal Cognitive Assessment (HI-MoCA) [39], an adaptation of the MoCA. Nonetheless, if the explanation of possible testing biases can partly account for cross-sectional associations between hearing impairment and low cognitive performances in the studies consisting of comparing the cognitive scores of hearing-impaired participants to non-impaired ones, such potential biases alone can hardly explain the now numerous studies reporting accelerated decline, since in longitudinal studies the participant’s performance is compared to his/her own over time and what is compared to a control group is the rate of decline, not inter-individual performance. Thus, although this hypothesis cannot be fully discarded, and possibly, for some of the results reported in the literature, may explain part of the association, other mechanisms probably come into play.

A major potential explanation relies on what is known as the “deprivation hypothesis” [19,40]. According to this hypothesis, hearing loss may lead to cognitive decline, and eventually dementia, because hearing impairment is suspected to affect brain integrity, as suggested by several MRI studies. For instance, a study conducted with 126 participants aged 56 to 86 years showed that the participants with hearing impairment exhibited accelerated brain atrophy (whole brain atrophy but more particularly in the right temporal lobe) compared to participants with normal hearing [41]. Consistently, another study conducted within the Rotterdam study on 2908 participants (mean age 65) reported that hearing impairment was associated with smaller total brain volume [42]. Moreover, a study showed that experimentally induced hearing loss in young mice caused a decreased performance in spatial working and recognition memory tasks at 6 months, providing evidence for a causal relationship between hearing loss and cognitive impairment [43].

An alternate view postulates that hearing loss may have a link with cognition because it leads to reduced physical activity, lower mood and poorer engagement in social and leisure activities. As shown by two studies [14,44], the strength of the association between hearing impairment and cognitive decline is reduced after statistically controlling for social participation variables. Moreover, low physical activity, depressive symptoms, as well as social isolation, have been reported as risk factors of cognitive decline and dementia in numerous studies [45,46,47,48,49,50]. Therefore, the link between hearing loss and cognition could be an indirect link mediated by reduced social and physical activity.

One mechanism that has been hypothesized to underlie the link between hearing loss and cognitive decline is related to the “cognitive load hypothesis” [51]. In the presence of hearing difficulty, the neural activity required to understand spoken language becomes more demanding if the brain is compelled to recruit additional neural populations to achieve accurate performance. The brain is assumed to constantly re-allocate attentional resources to help with deciphering and decoding the garbled auditory signal, which, in turn, may have a cognitive cost. In sum, cognitive impairment associated with hearing loss could be the result of devoting high cognitive resources to effortful sensory perception, to the detriment of other cognitive processes/brain areas [37].

Finally, the “common cause hypothesis” assumes the existence of a common factor that is responsible for the progressive degradation in physiological systems with aging. Cognitive as well as non-cognitive changes with advancing age are the result of multiple dysfunctions which are partly distinct and partly entangled. According to this hypothesis, both hearing loss and cognitive impairment are caused by common underlying processes such as generalized effects of the aging brain or age-related cerebrovascular disease. In its original view, the hypothesis stated that sensory acuity (including hearing and vision acuity) could be markers of global physiological integrity of the brain [13].

Although presented as alternatives, the different hypotheses proposed to explain the hearing–cognition relationship may not be mutually exclusive. On the contrary, it is more than likely that several mechanisms, or all of them, act in conjunction.

## 3. Treatment of Hearing Impairment in Older Adults and Impact on Cognitive Decline

### 3.1. Potential Benefits of Hearing Aids on Cognition

As mentioned above, hearing impairment is not only very frequent in older adults, it also has significant consequences on cognitive and mental health. Despite this, hearing loss in elders is largely underdiagnosed, and thus undertreated. Indeed, almost two-thirds of older people exhibiting hearing impairment do not use hearing aids [52]. There is some evidence, including evidence from randomized trials [53], for improved social functioning, communication and quality of life in older adults receiving hearing aids. Unfortunately, contrasting with the abundant literature reporting the consequences of hearing loss for cognitive function, there are few studies available reviewing the effects of hearing aids on cognition, even though a growing number of studies have investigated the question (see Table 1 for a summary of main studies).

One of the first studies addressing this question was conducted within the Health, Aging and Body Composition (Health ABC) cohort study [19], a US prospective epidemiologic survey which began in 1997–1998. The study relied on 1984 community-dwelling older adults, aged 77 on average, who were followed up for 6 years. The main result of this study was that hearing loss was associated with an accelerated cognitive decline measured with the Modified Mini-Mental State (3MS), a test providing a measure of global cognitive functioning. Nonetheless, as a secondary result, the study also reports slightly attenuated rates of cognitive decline in those participants who reported using hearing aids. However, possibly because of the relatively short follow-up period (i.e., 6 years) and the potential lack of statistical power, the result did not reach statistical significance.

An ancillary study was conducted in a subsample of the ARIC study, a prospective population-based cohort study of 15,792 participants recruited in 1987–1989 from four US communities and involving repeated measures of cognitive function including tests of episodic memory (delayed word recall test), language skills (word fluency test), and processing speed/attention (digit symbol substitution test). In this ancillary study on hearing impairment, audiometric testing, in addition to cognitive testing administered three times, was offered to 253 participants with an average age of 77 [54]. The main result was that participants with moderate and severe hearing impairments presented a higher estimated 20-year decline in memory and global function compared to those participants with normal hearing. Like in the previous study, information on whether participants used hearing aids or not was available, which led the authors to conduct a specific analysis on these participants. Among the 85 participants with moderate/severe hearing impairment, 43 reported using a hearing aid. The results showed that the estimated declines were greater in participants who did not wear a hearing aid than in those who did. Actually, the global decline in persons with moderate/severe hearing impairment who used hearing aids was only slightly greater than that observed in participants with normal hearing (estimated 20-year decline in a global cognitive composite score of −0.97 versus −0.90, respectively).

A cross-sectional analysis conducted within the English Longitudinal Study of Ageing (ELSA) population-based study supported the hypothesis of a positive impact of hearing aids’ use on cognition [44]. The study relied on a cross-sectional analysis of the data from wave seven of the ELSA study consisting of 7385 participants (with an average age of 67), of whom 3056 had mild hearing loss, 755 had severe hearing loss and 834 used a hearing aid. Cognitive functions were measured with tests of memory (10-word list immediate and delayed recall test) and executive function (verbal semantic fluency task). Hearing loss was associated with poorer cognitive scores. However, this association was evidenced only in the individuals who did not use hearing aids. For the subsample of respondents who used hearing aids, there was no evidence of association between hearing loss and cognition. Interestingly, the analysis involved an index of social isolation. After introducing this index in the statistical model, the results suggested that social isolation acted as a mediating factor, in particular in those participants who did not use hearing aids.

A single-arm interventional study was conducted in 34 older adults with hearing impairment who completed a depression scale (the geriatric depression scale) as well as the mini mental state examination (MMSE) test assessing general cognitive functioning before receiving hearing aids and 3 months after [55]. After 3 months of using a hearing aid, the study reported a significant improvement in scores of both depressive symptoms and cognitive function.

Based on these promising, although not fully convincing, results—due to lack of statistical significance [19], very limited sample sizes (43 participants using hearing aids in Deal et al.’s study [54]), cross-sectional design [44], or very-short follow-up (3 months in Acar et al.’s study)—an analysis conducted within the PAQUID study was designed to investigate more specifically the question of whether hearing aids’ use modulates long-term cognitive decline [14]. The PAQUID study is a French prospective population-based study involving, at baseline, 3777 participants aged 65 and over. The association of hearing loss, hearing aids’ use and cognitive decline was assessed in 3670 older adults followed-up for 25 years. At each follow-up visit of the study, numerous variables, including cognitive and functional measures, are collected by a trained psychologist. For those participants suspected to have cognitive disorders, a physician performed an additional medical assessment allowing confirmation of a clinical diagnosis of dementia and specifying the underlying etiology. Hearing loss was collected by a questionnaire assessing self-perceived hearing loss. At baseline, 137 subjects reported major hearing loss, 1139 reported moderate problems (i.e., difficulty following the conversation when several people talk at the same time or in a noisy background) and 2394 reported no hearing loss. Cognitive function was measured by MMSE, administered at each visit. The results showed that hearing loss was associated with lower MMSE scores at baseline and greater decline in the next 25 years of follow-up independently of age, gender and education. Such a greater decline was observed in the group of participants with hearing loss and not using hearing aids compared to the normal hearing group. In contrast, the group of participants using a hearing aid presented no significant difference in cognitive decline compared to the normal hearing group.

Interestingly, in a subsequent study conducted within the same population-based cohort (i.e., PAQUID study), these results were replicated on the risk of developing dementia [30]. Adjusting for age, gender and educational level, an increased risk of developing dementia was found for participants reporting hearing loss compared to participants reporting normal hearing. However, the association between hearing loss and risk of dementia revealed to be significant only in those participants who did not use hearing aids. This group presented a 21% increase in the risk of developing dementia in the next 25 years. Those with a hearing impairment who reported using hearing aids presented no increased risk of developing dementia. Notably, their risk of dementia was equivalent to that of participants reporting no hearing impairment. Similar results were found with the risk of presenting disability in both instrumental and basic activities of daily living in the 25 years of follow-up of the study, that is, a significantly increased risk in older adults with hearing loss not using hearing aids but no increased risk in those using hearing aids.

It is worth mentioning one study which reported slightly different results [56]. In an epidemiologic study conducted in the UK on 666 community-dwelling older adults with a hearing handicap (assessed with the hearing handicap inventory for the elderly; HHIE-S) seen at baseline, 5 and 11 years later, the results showed no difference at any timepoint in cognition measured with various tests (MMSE, trail making test, auditory verbal learning, digit-symbol substitution, verbal fluency), social engagement (hours per week spent in solitary activities), or mental health (SF-12 mental component) when comparing hearing-aid users to non-users, even though at the 11-year follow up, hearing aid users had a lower score of hearing handicap and better physical health.

Therefore, before building a clear conclusion on the effect of hearing aids’ use on cognitive decline, it is important to underline three important caveats: (1) the scarce number of studies, in particular longitudinal ones; (2) one study providing contrasting results; and (3) the absence of results gathered from interventional clinical studies, in particular randomized controlled trials providing a full demonstration. Nonetheless, the available data globally support the hypothesis that hearing aids have a positive impact on long-term cognition in older adults suffering from hearing loss.

### 3.2. Cochlear Implants and Cognitive Outcomes in Older Adults

With a more severe to profound degree of hearing loss, conventional hearing aids may increase sound detection without substantially improving speech perception in communication due to central auditory processing deficits. Cochlear implantation may be a therapeutic option for older adults with severe to profound hearing loss who continue to experience poor word discrimination despite appropriately fitted hearing aids [57]. Nonetheless, cochlear implantation typically requires surgery, and a period of training and therapy after surgery. In the US, only 5% to 10% of adult cochlear implant candidates have received cochlear implants, even though Medicare and many insurance carriers contribute financially to the medical cost [58,59]. The average delay between onset of severe to profound hearing loss and the receipt of a cochlear implant is approximately 10 years [60].

Studies looking at the effects of cochlear implantation on cognitive outcomes in elders are very few (see Table 2 for a summary of main studies). A review was performed to determine the status of the literature on the potential influence of cochlear implantation on cognition in the older adult population [61]. More than 3000 articles related to cochlear implants, cognition, and older adults were initially reviewed. However, after applying the inclusion criteria (i.e., (1) study population including adults >65 years, (2) intervention with cochlear implantation, and (3) cognition as the primary outcome measure of implantation), only three studies could be included in the review. A study assessed neuropsychological status of 13 subjects aged 23 to 67 (mean age 48) with single-channel cochlear implants [62]. Cognition was measured with subtests of the Halstead–Reitan neuropsychological test battery and the Wechsler–Bellevue Form II test. Pre-implantation assessment was not available, so changes in cognition pre- versus post-implantation could not be measured. Post-implant test results showed that 11 participants scored within normal limits and two showed impaired cognitive scores. Another study analysed the data from 46 post-lingually deafened adults aged 19 to 75 years (mean age 48) who underwent cochlear implantation with a single-channel device [63]. The patients performed an extended cognitive battery (Wechsler Adult Intelligence Scale assessing general cognitive ability, Graham–Kendall Memory for Designs measuring immediate recall, Trail Making Test assessing executive functions and Bender Visual Motor Gestalt and Interference Test for visual motor functions) before implantation, one year post-implantation, and, for some patients, 2 years after implantation. The results showed that there was no damage in cognitive function post-implantation and that some individuals showed improvement in several of the cognitive tests. A third study examined the psychological status pre- and post-implantation of 30 adults with profound post-lingual hearing loss aged 14 to 80 years (mean age 49) who received a multi-channel cochlear implant [64]. Cognitive assessment included the Wechsler Adult Intelligence Scale-Revised. The results showed patients’ improvement in communication abilities early after implantation. However, the small number of subjects and lack of long-term follow-up did not allow conclusions to be drawn about changes in cognition after implantation. Based on these three studies, the conclusion of the review [61] was that, while many publications have shown that cochlear implants improve speech perception, social functioning, and overall quality of life, there is insufficient data to allow conclusions regarding the effects on cognition.

A more recent review examined the results of six studies, relying on a total of 166 patients [72]. Five of these studies reported improvements in cognition after implantation [65,66,67,68,69] and one study did not observe significant changes [70]. Although the results are globally promising, the authors underlined the numerous biases of such studies, including small sample sizes, lack of control group in some studies, lack of control of practice effects, inappropriate statistical methods and lack of long-term follow-up measures. For these reasons, even though this review included more studies than the previous one, the conclusion of the authors was that the existing literature does not provide conclusive evidence of improved cognitive outcomes after cochlear implantation in older adults.

After the publication of these two reviews, a clinical study examined the effect on cognition of cochlear implantation on 59 adults aged 61–89 years with severe-profound hearing loss and no previously diagnosed or suspected cognitive impairment [71]. At 18 months, significant benefits of cochlear implants were observed in terms of speech perception, communication ability, and quality of life. Improvement in executive function (measured with the Groton Maze Learning Test of the CogState battery) was found in a subsample of participants, while cognitive function remained stable for other ones. The follow-up of the patients is still ongoing, so further follow-up will reveal the effects of cochlear implant intervention on all outcomes, and in particular whether it can delay cognitive decline.

## 4. Conclusions and Perspectives

The first conclusion of this review is that there is a growing interest in research on this topic, which is good news since it reflects a collective enlightenment regarding hearing issues in older age. In recent years, research evidencing its major consequences for cognitive and mental health has been a determinant contributing to changes in the view that hearing loss in older age is a banal and negligible condition.

In the report of the Lancet Commission on “dementia prevention, intervention and care” [73], one of the key messages is the need to be ambitious in terms of prevention. Using population attributable fractions, the authors estimate that as many as 35% of dementia cases could be prevented by targeting nine modifiable risk factors: education, hypertension, obesity, smoking, depression, physical inactivity, diabetes, social isolation and hearing loss. Middle-life hearing loss would account for 9% of dementia cases. Therefore, there is an urgent need to have clear knowledge of the potential benefits of treating hearing loss on cognition.

Regarding cochlear implants, if there is a link between hearing loss and cognitive decline, the elderly population experiencing the most severe degree of auditory deprivation would be an obvious candidate group for cochlear implants, with potential benefits for mental and cognitive heath. However, the question of whether cochlear implantation helps improve cognition is far from clear, due to the scarce number of studies and the many biases which may explaining the positive results reported in the available publications.

Regarding hearing aids, the literature is slightly more convincing, even though building an irrefutable conclusion on the positive effect of hearing aids’ use on cognitive decline is difficult. In particular, the absence of results gathered from randomized controlled trials providing a full demonstration that the use of hearing aids reduces cognitive decline and/or postpones dementia onset calls for caution. However, regarding the last point, it may be important to remember that cognitive decline, as well as dementia, are a slowly progressive phenomena that can only be captured over a long timescale of several years or decades. Thus, long-term epidemiologic studies, although observational, may provide valuable knowledge, since the protective effects of hearing aid use may be hardly observable in clinical trials involving small samples of participants or a short-duration follow-up. Notably, the ongoing ACHIEVE study, a large trial on 850 older adults, will be the first randomized trial to determine the efficacy of a best-practices hearing intervention (compared to a successful aging intervention) on reducing cognitive decline in older adults with hearing loss [74]. Awaiting these results, and keeping in mind the previously mentioned caveats, the available data globally support the hypothesis that hearing aids’ use positively impacts long-term cognition in older adults suffering from hearing loss, but definitely call for more research to ascertain such a statement and provide more information on the delay at which the benefits can be observable, the daily frequency of hearing aids use required, and many other questions that remain fully open.

By highlighting the limitations of the existing literature, this review will hopefully stimulate more necessary research, as preventing cognitive decline and dementia is undoubtedly a major challenge for our society, and treating hearing loss is a promising and “accessible” strategy that should be seen as a component in multidimensional interventions. Indeed, even though this review was intentionally limited to the benefits of treating hearing loss, interventions addressing a wider range of risk factors, such as lifestyle factors and nutrition [75], should be recommended to slow down the vicious circle of hearing loss and cognitive impairment.

## Figures and Tables

**Table 1:** Main studies assessing the association between hearing aid use in older adults and cognitive outcomes.

Study	Cross-Sectional or Prospective Design (and Length Follow-Up)	Sample Size	Cognitive Tools	Main Result
Ray et al. 2018 [44]	Cross-sectional	7385	10-word list immediate and delayed recall test, verbal fluency task	Higher cognitive scores in HA users
Lin et al. 2013 [19]	Prospective: 6-year follow-up	1984	3MS	No significant difference
Deal et al. 2015 [54]	Prospective: 20-year follow-up	253	Delayed word recall test, verbal fluency task, DSST	Lower decline in HA users in a global composite score
Acar et al. 2011 [55]	Prospective: 3-month follow-up	34	MMSE	Lower decline in HA users
Amieva et al. 2015 [14]	Prospective: 25-year follow-up	3670	MMSE	Lower decline in HA users
Dawes et al. 2015 [56]	Prospective: 11-year follow-up	666	MMSE, TMT, auditory verbal learning, DSST, verbal fluency task	No significant difference

HA, Hearing aids; 3MS, Modified Mini Mental State Examination; DSST, Digit symbol substitution test; MMSE, Mini Mental State Examination; TMT, Trail Making Test.

**Table 2:** Main studies assessing the association between cochlear implantation in older adults and cognitive outcomes.

Study	Length Follow-Up after Cochlear Implantation	Sample Size	Cognitive Tools	Main Result
Crary et al. 1982 [63]	1 year	46	WAIS, Graham–Kendall Memory for Designs, TMT, Bender Visual Motor Gestalt	Improved performance in a subsample of participants by descriptive data only
Castiglione et al. 2015 [65]	1 year	15	MOCA	Improved performance
Mosnier et al. 2015 [66]	1 year	94	MMSE, Five-Word Test, Clock drawing test, d2 test of attention, TMT	Improved performance
Cosetti et al. 2016 [67]	3.7 years in average	7	WAIS, TMT, controlled oral word association test, Boston naming test, RBANS	Improved performance reported in some participants by descriptive data only
Ambert-Dahan et al. 2017 [68]	1 year	18	CODEX, MOCA	Improved performance reported in some participants by descriptive data only
Jayakody et al. 2017 [69]	1 year	39	CANTAB	Improved performance
Sonnet et al. 2017 [70]	1 year	16	MMSE, Rey complex figure test, TMT, Five-Word Test, oral naming test DO80	No difference
Sarant et al. 2019 [71]	1.5 year	59	Groton Maze Learning Test from CogState battery	Improved performance in a subsample of participants

WAIS, Wechsler abbreviated scale of intelligence; TMT, trail making test; MOCA, montreal cognitive assessment; MMSE, mini mental state examination; RBANS, repeatable battery for the assessment of neuropsychological status; CODEX, cognitive disorders examination; CANTAB, Cambridge neuropsychological test automated battery.
